# Factors associated with severe dry eye in primary Sjögren’s syndrome diagnosed patients

**DOI:** 10.1007/s00296-018-4013-5

**Published:** 2018-03-20

**Authors:** Mónica Fernandez Castro, Carlos Sánchez-Piedra, Jose Luis Andreu, Víctor Martínez Taboada, Alejandro Olivé, Jose Rosas, On behalf of SJOGRENSER Group, part of the Spanish Society of Rheumatology Systemic Autoimmune Diseases Study Group (EASSER)

**Affiliations:** 1 Rheumatology, Hospital Universitario Infanta Sofía, Avenida de Europa 34, 28703 San Sebastian de los Reyes, Madrid Spain; 2 Research Unit, Spanish Society of Rheumatology, Madrid, Spain; 3 Rheumatology, Hospital Universitario Puerta de Hierro Majadahonda, Madrid, Spain; 4 Rheumatology, Hospital Marqués de Valdecilla, Santander, Spain; 5 Reumatology, Hospital Germans Trias i Pujol, Barcelona, Spain; 6 Reumatology, Hospital Marina Baixa, Villajoyosa, Spain

**Keywords:** Dry eye syndromes, Sjögren’s syndrome, Joints, Schirmer’s test

## Abstract

**Introduction:**

Primary Sjögren’s syndrome (pSS) is an autoimmune disease, characterized by lymphocytic infiltration of exocrine glands and other organs, resulting in dry eye, dry mouth and extraglandular systemic findings.

**Objective:**

To explore the association of severe or very severe dry eye with extraocular involvement in patients diagnosed with primary Sjögren’s syndrome.

**Methods:**

SJOGRENSER registry is a multicenter cross-sectional study of pSS patients. For the construction of our main variable, severe/very severe dry eye, we used those variables that represented a degree 3–4 of severity according to the 2007 Dry Eye Workshop classification. First, bivariate logistic regression models were used to identify the effect of each independent variable on severe/very severe dry eye. Secondly, multivariate analysis using regression model was used to establish the independent effect of patient characteristics.

**Results:**

Four hundred and thirty-seven patients were included in SJOGRENSER registry; 94% of the patients complained of dry eye and 16% developed corneal ulcer. Schirmer’s test was pathological in 92% of the patients; 378 patients presented severe/very severe dry eye. Inflammatory articular involvement was significantly more frequent in patients with severe/very severe dry eye than in those without severe/very severe dry eye (82.5 vs 69.5%, *p* = 0,028). Inflammatory joint involvement was associated with severe/very severe dry eye in the multivariate analysis, OR 2.079 (95% CI 1.096–3.941).

**Conclusion:**

Severe or very severe dry eye is associated with the presence of inflammatory joint involvement in patients with pSS. These results suggest that a directed anamnesis including systemic comorbidities, such as the presence of inflammatory joint involvement or dry mouth in patients with dry eye, would be useful to suspect a pSS.

## Introduction

Primary Sjögren’s syndrome (pSS) is the second most common autoimmune disease, of unknown etiology and slow progression, with a prevalence of 0.1–5% in the general population. It affects predominantly the female sex and is characterized by lymphocytic infiltration of exocrine glands and other organs, resulting in dry eye and dry mouth and extraglandular systemic findings, such as pain, myalgia or polyarthralgia, among others [1]. The condition has a marked negative impact on health-related quality of life and social functioning [2].

Dry eye is one of the most common problems encountered by eye care practitioners globally. In the USA alone, an estimated 16.4 million adults aged ≥ 18 years have dry eye disease (DED) [3]. DED is a multifactorial disease with a variety of risk factors identified, including the association of several systemic diseases such as pSS. In 2007, the International Dry Eye Workshop (DEWS) compiled an evidence-based review of the methods used to evaluate, diagnose and manage DED [4]. In 2016, the Tear Film and Ocular Surface Society (TFOS) DEWS II [5] has identified two predominant classes of dry eye: aqueous–deficient and evaporative [6]. Aqueous-deficient dry eye is subcategorised into pSS-related dry eye and non-pSS dry eye. Of individuals with a significant aqueous deficient dry eye, 10% are likely to have Sjögren síndrome [7]. In harmony of the classification criteria by American-European consensus group (AECG) [8], pSS consists of the occurrence of aqueous deficient dry eye syndrome in combination with symptoms of dry mouth, in the presence of autoantibodies, evidence of reduced salivary secretion and with a positive focus score on minor salivary gland biopsy.

Because dry eye is one of the most common symptoms of pSS, patients often first seek care from eye care providers, who can potentially play a key role in reducing the time from symptom onset to diagnosis. In SJOGRENSER registry the delay in diagnosis from the onset of the first symptoms is substantial, with a median age of the first symptom of 47 years and a median age at diagnosis of 50 years [9]. Nevertheless, screening is challenging, as there is currently no universal standard regarding which dry eye patients should undergo a comprehensive evaluation for pSS, there is limited evidence regarding specific ocular signs that in isolation can reliably distinguish pSS-related from non-SS-related dry eye. The aim of this study is to explore factors associated with severe or very severe dry eye in patients with pSS. Identification of variables that are associated with the development of pSS may contribute to earlier diagnosis and treatment of the disease, and ultimately better long-term outcome.

## Methods

### Study design and population

#### Study design

This is a descriptive cross-sectional study of pSS patients fulfilling 2002 AECG criteria [8], from 33 Spanish rheumatology departments participating in SJOGRENSER study, a Systemic Autoimmune Disease Group Project of the Spanish Society of Rheumatology. The objectives and methodology of the SJOGRENSER Registry have been published previously [9].

#### Study population

Four hundred and thirty-seven patients with pSS were included in the SJOGRENSER Registry. To avoid any selection bias, patients were selected by randomization from an anonymized list provided by every unit. Data were collected by reviewing clinical records and interviewing patients. The exclusion criteria were also based on the AECG criteria; in addition, we excluded patients younger than 18 years’ old or individuals unable to provide informed consent. This registry was performed between 2013 and 2014 over a 12-month period of time. An online monitored control of all the information and an on-site monitored control of a percentage of centers randomly selected were used to clarify all inconsistencies, missing values, and discrepancies. A signed informed consent was obtained from the patients and the ethics committees of the hospitals approved the study.

#### Variables

The sociodemographic variables from SJOGRENSER registry included for this article were age, sex, time of evolution of disease and clinical variables like systemic manifestations (Table 1) and the presence of ocular symptoms included in the subjective AECG criteria: (1) Have you had daily, persistent, troublesome dry eyes for more than 3 months?, (2) Do you have a recurrent sensation of sand or gravel in the eyes? (3) Do you use tear substitutes more than 3 times a day? [10]. We also included dry eye complications like corneal ulcers and, serological parameters like ANA (antinuclear antibodies), RF (Rheumatoid Factor) and Anti-Ro/Anti-La, and labial salivary gland (LSG) biopsy; in our cohort, we collected the Chisholm and Mason grades (considering as pathological 3 and 4 grades, since they correspond to one or more lymphocyte infiltration foci respectively, focus score ≥ 1). The standardized objective measurement for dry eye collected were Schirmer’s *I* test (pathological according to AECG criteria:≤5 mm/5 min) and ocular stains. Data from pSS related symptoms and activity index, were collected using the EULAR Sjögren’s Syndrome Patient Reported Index (ESSPRI) [11] and EULAR Sjögren’s Syndrome Disease Activity Index (ESSDAI) [12]. The pharmacological and surgical therapeutic options included in this article were autologous sera, contact lenses, punctual occlusion, Stenon conduit bypass and palpebral cleft reduction.

**Table 1:** Baseline characteristics of SjögrenSER patients according to the severity in the dry eye

Variables	Dry eye not severe (*n* = 32)	Severe/very severe dry eye (*n* = 378)	*p*
Age at diagnosis, mean (SD)	49.33 (13.9)	50.56 (12.7)	0.195
Sex, women *n* (%)	57 (96.6)	359 (95.0)	0.585
Index			
ESSPRI, mean (SD)	5.20 (2.5)	5.28 (2.3)	0.980
ESSDAI, mean (SD)	5 (7)	5 (5)	0.854
Systemic manifestations			
Inflammatory articular involvement *n* (%)	41 (69.5)	312 (82.5)	0.028*
Pulmonar involvement *n* (%)	6 (10.3)	37 (9.8)	0.895
Renal involvement *n* (%)	6 (10.3)	33 (8.7)	0.688
Central nervous system involvement *n* (%)	5 (8.6)	29 (7.7)	0.802
Glandular inflammation *n* (%)	21 (35.6)	121 (32.0)	0.585
Peripheral nervous system involvement *n* (%)	5 (8.6)	34 (9.0)	0.926
Digestive involvement *n* (%)	11 (18.6)	48 (12.7)	0.214
Hematologic involvement *n* (%)	31 (52.5)	213 (56.3)	0.584
Pathological labial salivary gland biopsy (focus score ≥ 1) *n* (%)	26 (44.1)	107 (28.3)	0.014*
Analysis			
ANA + *n* (%)	58 (98.3)	366 (96.8)	0.534
RF + *n* (%)	39 (72.2)	245 (65.7)	0.341
AntiRo + *n* (%)	58 (98.3)	351 (92.9)	0.112
AntiLa + *n* (%)	42 (71.2)	251 (66.4)	0.467

* *p* < 0.05

To define our main variable, “severe or very severe dry eye”, we included the subjective and objective criteria of the TFOS DEWS I consensus classification of 2007 [4]. They recommend dry eye treatments based on the level of disease severity (LDS). The severity grading scheme contains 4 LDS based on signs and visual symptoms, among them: conjunctival injection, conjunctival staining, corneal and tear signs, lid and meibomian glands, tear break-up time and Schirmer score. Not all of these variables were collected in our registry, so for the construction of our main variable, we used those variables present in our cohort that represented a degree 3 or 4 of severity (severe or very severe dry eye, respectively) according to the TFOS DEWS classification for dry eye. Those variables were: the pathological Schirmer score (corresponds with a 3 LDS), corneal ulcers (correspond with a 4 LDS), autologous sera and contact lenses (corresponds with a 3 LDS) and, Stenon conduit bypass and palpebral cleft reduction (corresponds with a 4 LDS).

Finally, in those patients with dry eye (any positive response to the 3 dryness questions included in the AECG criteria), the selected variables to define “severe or very severe dry eye” were: Schirmer score and/or corneal ulcers and/or autologous sera and/or contact lenses and/or Stenon conduit bypass and/or palpebral cleft reduction. We did not consider ocular stains because of the high percentage of losses in the collection of this variable by researchers. Blepharitis was not selected because it appears on the dry eye severity scale at all levels; the frequency of appearance of blepharitis is not collected in the SJOGREN-SER cohort. Punctual occlusion has not been included because in SJOGREN-SER registry there is no difference between transient punctual occlusion (2 LDS) and permanent (4 LDS).

### Statistical analysis

We developed descriptive analyses, using bivariate correlations and regression analysis to model the association between “severe or very severe dry eye” and clinical variables recorded in SJOGRENSER. The descriptive analysis consists of median and interquartile ranges, which were used to describe numeric variables. Proportions were used for categorical variables. Chi-Square test was used for categorical variables and Kruskal Wallis test for numeric variables. Statistical significance was considered as a *p* < 0.05.

First, bivariate logistic regression models were used to identify the effect of each independent variable on severe or very severe dry eye. Second, multivariate analysis using regression model was used to establish the independent effect of patient characteristics associated with the dependent variable. The model included odds ratio (OR), 95% confidence intervals and associated statistical significance. Sex, age and ESSDAI score were considered as adjustment variables in the final regression model.

The analysis was performed using SPSS 21.0 for Windows (SPSS Inc., Chicago, Illinois, USA).

## Results

Four hundred and thirty-seven patients were included in SJOGRENSER registry [female gender 95%; median age 58 (50.02–67.98) years]. Mean time of evolution of the disease in the cohort was 8.3 years. All patients were ANA positive, 94% were AntiRo +, 67% were AntiLa +, and 65% RF positive. ESSDAI mean score was 2 (0–4, P25–P75) and the median in ESSPRI index was 5.3 (p25–p75, 3.67-7) in the full registry. 94% of the patients in SJOGRENSER registry complained of daily, persistent, troublesome dry eye, 92% had sensation of sand and, 16% developed corneal ulcer.

In the full cohort, Schirmer’s test was performed in 402 patients and was pathological in 371 patients (92%). Rose Bengal staining test was performed in 144 patients, green lissamine and fluorescein staining tests were performed in 19 and 81 patients respectively. LSG biopsy was obtained in 193 patients, 133 of them fulfilling grades 3 or 4 of Chisholm-Mason classification (69%). The use of autologous sera was 14%, contact lenses 2%, Stenon conduit bypass 0.23% and palpebral cleft reduction 0.23%.

378 patients (86.5%) presented severe or very severe dry eye; 95% were women and the median age was 50 years. Mean time of evolution of disease was 8.51 years. ESSDAI mean score in this subgroup was 5 and the median in ESSPRI index was 5.28. 312 patients developed inflammatory articular involvement, according to the definition of the ESSDAI index: all of them reported inflammatory joint pain and 132 also presented arthritis. The baseline characteristics of the patients according to the severity of the dry eye are shown in Table 1. Inflammatory articular involvement was significantly more frequent in patients with severe/very severe dry eye (82.5 vs 69.5%, *p* = 0.028) (Table 1). In the descriptive analysis, the pathological LSG biopsy was more frequent in individuals with non-severe dry eye, however, in the multivariate analysis, no significant association was found between this variable and the non-severe dry eye. In the bivariate (Table 2) and in the multivariate analysis (Table 3), inflammatory joint involvement was a factor associated with severe/very severe dry eye, with an OR of 2.079 (95% CI 1.096–3.941) adjusted by sex, age, time of evolution of the disease and ESSDAI score (Table 3). No relationship was found between severe/very severe dry eye and the rest of extraglandular manifestations (Table 1).

**Table 2:** Bivariate analysis results

Variables	Wald	OR	Confidence interval
Age at diagnosis	0.462	1.007	0.986–1.029
Sex, women	0.295	0.663	0.150–2.923
Time of evolution of SSp	2.291	0.136	0.99–1.085
Index			
ESSPRI	0.061	1.015	0.902–1.143
ESSDAI	0.014	0.997	0.950–1.047
Systemic manifestations			
Inflammatory articular involvement	5.423	2.075	1.123–3.837
Pulmonar involvement	0.018	0.940	0.378–2.338
Renal involvement	0.161	0.829	0.331–2.075
Central nervous system involvement	0.063	0.881	0.327–2.375
Glandular inflammation	0.298	0.852	0.479–1.578
Peripheral nervous system involvement	0.009	1.048	0.392–2.798
Digestive involvement	1.524	0.635	0.308–1.306
Hematologic involvement	0.300	1.166	0.673–2.021
Pathological labial salivary gland biopsy	5.835	0.501	0.286–0.878
Analysis			
ANA +	0.374	0.526	0.067–4.121
RF +	0.900	0.736	0.391–1.386
AntiRo +	2.116	0.224	0.030–1.681
AntiLa +	0.527	0.800	0.438–1.461

Factors associated with severe dry eye in SSp

**Table 3:** Multivariate analysis results

Variables	Wald	OR	Confidence interval
Age at diagnosis	1.901	1.016	0.993–1.039
Sex, women	1.310	0.252	0.039–2.342
Time of evolution of the disease	3.323	1.046	0.997–1.098
Index			
ESSDAI, mean (SD)	0.476	0.982	0.934–1.033
Systemic manifestations			
Inflammatory articular involvement	5.026	2.079	1.096–3.941
Labial salivary gland biopsy			
Focus score ≥ 1	2.451	0.519	0.229–1.179
Unknown	0.039	1.079	0.504–2.309

Factors associated with severe dry eye in SSp

## Discussion

The aim of this study was to identify factors associated with severe or very severe dry eye in patients with pSS. We have found two main findings, first we have found a high prevalence of severe or very severe dry eye in our population of patients with pSS; secondly, we have identified a strong association between severe dry eye and inflammatory articular involvement. Taking into account that the majority of pSS patients first seek medical care for dry eye symptoms, that dry eye disease is highly prevalent in the general population, and that pSS evaluations are costly, complex, and time-consuming, it is of great interest to be able to draw up a list of factors associated with severe dry eye, often developed by pSS patients. At least two ophthalmologic methods (Schirmer test and ocular staining) are recommended for the evaluation of dry eye. Not all involved patients in this cohort had these two tests, so in this sample dry eye is defined as severe/very severe with other included criteria. We examined the associations of an individual dry eye test result (Schirmer test), complications and treatments used in severe dry eye in pSS patients, with extraocular characteristics for pSS.

Other authors have also found a relationship between joint involvement and dry eye. Paulsen et al. [13] investigated the prevalence of dry eye (14.5%) and identified independent risk factors in 3285 patients. In the multivariable model, dry eye symptoms were associated with arthritis (OR 2.14) in patients younger than 50 years. A limitation of the Paulsen’s study was that only a few participants reported a history of being diagnosed by their doctor as having SS (Sjögren’s syndrome). This same cohort was examined years ago by Moss et al. (1993–1995) [14], also finding at that time, an association between dry eye and arthritis (OR 1.91).

Roh et al. [15] tried to identify systemic comorbidities in patients with dry eye syndrome in South Korea (*n* = 17,364), with a prevalence of dry eye of 10.4%. Degenerative arthritis (OR 1.57) and rheumatoid arthritis (OR 1.44) were associated with a significantly higher prevalence of dry eye. One limitation of Roh’s study, regarding the topic of interest of this article, is that it is not known whether the patients of this cohort were evaluated for pSS in a specific way.

Other authors have found a relationship between dry eye and other clinical or serological parameters. Liew et al. [7] reported a prevalence of SS in patients with clinically significant aqueous-deficient dry eye (ADDE) of 11.6%. They found that patients with SS had significantly worse conjunctival and corneal staining, Schirmer test, and symptoms compared with patients without SS; SS was also significantly more likely to occur in patients with ANA and RF.

This finding was also noted in the studies of Whitcher and colleagues and Lim and collaborators [16, 17]; both reported that patients with SS were more likely to be positive for RF and ANA. These authors belong to the SICCA group (Sjögren’s International Collaborative Clinical Alliance) [18]; they evaluated 920 participants and found that in the keratoconjunctivitis sicca (KCS) group, the median OSS (Ocular Staining Index, contemplated in SICCA classification criteria 2012) was 5 compared to 9 among those with SS-KCS (*p* < 0.0001). A statistically significant difference with respect to the Schirmer test reflected a higher level of severity in the SS-KCS than in the KCS-only group. In 2012, Chung et al. also published that serum anti-La antibody, serum anti-Ro antibody and tear IL-17 were likely strongly involved in the clinical severity of KCS in patients with pSS [19]. All patients in the SJOGRENSER registry were ANA + and the majority were AntiRo +, so no differences could be established between groups using these serological parameters.

From the previous cohort (SICCA group), Bunya et al. [20] described how to differentiate those patients with dry eye with a greater suspicion of SS in a cohort of 3514 individuals recruited with SS or possible SS, evaluating the relationship that exists between ocular surface tests and typical extraocular manifestations of SS; they found that those with an abnormal Schirmer I test or positive ocular stain present a significantly higher probability of having a positive LSG biopsy or a positive serology.

Many other authors conclude and demonstrate a high frequency of associated SS in patients with dry eye [21]. Martinez et al., in 2016 [22], studied the frequency and risk factors of dry eye among patients in Mexico. The frequency of severe dry eye symptoms was found to be between 43 and 30%. Dry mouth was a risk factor significantly associated with increased dry eye symptoms, aqueous tear deficiency, and corneal staining and, the presence of arthritis was a risk factor associated with meibomian gland dysfunction.

As we have seen, abnormal Schirmer tests are of vital importance when SS is suspected in patients with dry eyes. The evaluation of the symptoms is essential in the assessment of dry eye, however, the standard procedure in the usual clinical practice for DED diagnosis includes the performance of at least one objective test [23]. The possible diagnostic tools in the evaluation of tear film disorders are multiple, however, the preferred ones are patient history/dry eye questionnaires, tear break-up time, ocular surface staining and Schirmer test [24, 25]. Sullivan et al., in 2014, concluded that the information on the ocular surface provided by each test is different, and demonstrated that for the diagnosis and treatment of DED not only the symptoms of the patient can be taken into account [26].

With regard to the lack of a gold-standard for the severe dry eye diagnosis, the multinational ODISSEY European Consensus Group, formed by ophthalmologists from different countries, established in 2014 an algorithm to identify the most relevant tools in the evaluation of the ocular surface and therefore its severity. This panel recommended that only two criteria, evaluation of symptoms and evaluation of the ocular surface with fluorescein staining, were adequate to assess severity in most patients. Not forgetting that this article focuses exclusively on patients with pSS, the authors of this group also described in their study that the Schirmer test is the quantitative test available to measure the lacrimal flow and therefore the maximum capacity of secretion of the lacrimal glands, key dysfunction in this disease; they added that this method is well established, easy to use, commonly accepted and available, safe and efficient and usually well tolerated (except in the severe DED) [27].

Our findings suggest that inflammatory articular involvement is significantly associated with severe dry eye in pSS patients, an assessment that can be made easily with a simple anamnesis like “do you have arthritis or inflammatory joint pain?”. The ESSDAI definition of inflammatory joint pain is “pain in hands/wrists/ankles and/or feet and stiff in the morning for at least 30 min” and the ESSDAI definition of arthritis is “joint inflammation”. pSS patients with inflammatory articular involvement have twice risk of severe dry eye in pSS patients compared to those who have no inflammatory joint involvement in our cohort. For ophthalmologists, a more careful interview for systemic comorbidities in patients with dry eye, like the presence of inflammatory articular involvement, dry mouth or other systemic manifestations, is possibly much easier, non-aggressive, faster and less expensive than performing other actions such as a blood test in search of specific antibodies or perform a minor salivary gland biopsy. The results of this study support calling for an increased index of suspicion for ophthalmologists caring for dry eye, and the recommendation that the presence of SS should be assessed in all patients with clinically significant DED. It is also of great interest that Schirmer test is an easy and a critical test that should always be included when screening dry eye patients to determine whether a further evaluation for pSS is warranted.

**Fig. 1:**
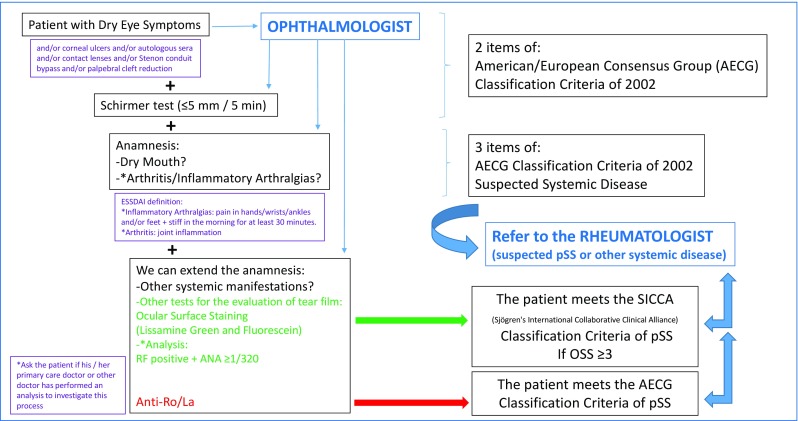
Algorithm for suspected diagnosis of pSS, based on the use of the Schirmer test and an anamnesis guided to the patient with dry eye

Undoubtedly, the typical antibodies of pSS and the LSG biopsy are two very important characteristics of pSS patients. However, they are tests that demand greater effort, cost, complexity, and invasion for the patient. An adequate anamnesis guided by the ophthalmologist is simple and fast and can give a lot of important information suspicious of pSS in the patient with severe dry eye. Therefore, we suggest a simple algorithm for suspected diagnosis of pSS, based on the use of the Schirmer test and an anamnesis guided to the patient with dry eye, so that the ophthalmologist can make an initial screening and know which patient with dry eye should be referred to the rheumatologist (Fig. 1). This algorithm should be considered as a proposal pending of consensus with more experts, ophthalmologists, and rheumatologists, and validated in the future with an ad hoc project.

A possible limitation of our study was the small number of patients in the non-severe dry eye group compared with the severe/very severe dry eye group (32/378); this can be explained by the high number of patients with dry eye (94%) and, by the high number of patients with pathological Shirmer test performed (371/402, 92%) (score equivalent to a severe dry eye); 85% of the patients recruited in this cohort (371/437) had severe/very severe dry eye at the time of inclusion in SJOGRENSER registry because they had a Schirmer test ≤ 5 mm. Another possible limitation could have been the failure to be able to do a complete study evaluating other objective tests of ocular dryness, such as ocular staining, due to the high percentage of losses in this variable.

In summary, for ophthalmologists, asking about the presence of inflammatory articular involvement and dry mouth is possibly much easier, non-aggressive, faster and less expensive, than performing other actions such as a blood test in search of specific antibodies or perform a minor salivary gland biopsy. A large prospective study is needed to estimate the association of other factors with dry eye. The possibility of a screening algorithm involving both ophthalmologist and rheumatologist should be tested in future studies. If a pSS is suspected, the ophthalmologist could refer the patient to the rheumatologist to complete the tests that the patient needs and for a right follow-up and systematic treatment, always in collaboration with the ophthalmologist, for multidisciplinary control of dry eye.
